# Biologic interactions determining geographic range size: a one species response to phylogenetic community structure

**DOI:** 10.1002/ece3.959

**Published:** 2014-02-27

**Authors:** Leonel Herrera-Alsina, Rafael Villegas-Patraca

**Affiliations:** 1 Posgrado en Ciencias Biológicas, Universidad Nacional Autónoma de MéxicoApartado Postal 27-3, CP 58089, Morelia, Michoacán, México; 2 Centro de Investigaciones en Ecosistemas, Universidad Nacional Autónoma de MéxicoApartado Postal 27-3, CP 58089, Morelia, Michoacán, México; 3 Instituto de Ecología, A. C.Carretera antigua a Coatepec 351, El Haya, Xalapa, 91070, Veracruz, Mexico

**Keywords:** Avian communities, competition intensity, distribution area, *Peucaea* sparrows, phylogenetic similarity

## Abstract

Range size variation in closely related species suggests different responses to biotic and abiotic heterogeneity across large geographic regions. Species turnover generates a wide spectrum of species assemblages, resulting in different competition intensities among taxa, creating restrictions as important as environmental constraints. We chose to adopt the widely used phylogenetic relatedness (NRI) measurement to define a metric that depicts competition strength (via phylogenetic similarity), which one focal species confronts in its environment. This new approach (NRI_focal_) measures the potential of the community structure effect over performance of a single species. We chose two ecologically similar *Peucaea* sparrows, which co-occur and have highly dissimilar range size to test whether the population response to competition intensity is different between species. We analyzed the correlation between both *Peucaea* species population sizes and NRI_focal_ using data from point counts. Results indicated that the widespread species population size was not associated with NRI_focal_, whereas the population of restricted-sized species exhibited a negative relationship with competition intensity. Consequently, a species' sensitivity to competition might be a limiting factor to range expansion, which provides new insights into geographic range analysis and community ecology.

## Introduction

Biologic interactions are fundamental to the understanding of ecological patterns and processes. Webb et al. (2002) set the framework of phylogenetic community ecology to elucidate the effect of ecological interactions depicted via phylogenetic structure, that is, the degree of relatedness among species forming an assemblage. This approach is strongly supported by the tested observation of the inheritance of niche-related traits from ancestors, termed niche conservatism (Wiens et al. 2010; for birds see Lovette and Hochachka 2006), which reinforces the concept of a link between phylogenetic distance and ecological similarity reflected in behavioral (Houle 1997) and life-history traits (Burns and Strauss 2011). Consequently, demonstrating statistical support for phylogenetic and community assemblage patterns (i.e., clustering or evenness) suggests a potential process exists structuring species assemblages (Webb et al. 2002). Questions addressed under this method have resolved several issues related to ecology (ecosystem stability Cadotte et al. 2012; species lost and climate change, Willis et al. 2008), providing useful insights into the internal structure of phylogenetic and ecological relationships (Lovette and Hochachka 2006, Gómez et al. 2010). For example, Graham et al. (2009) demonstrated that biologic interactions among hummingbird species represented the leading factor in tropical lowland community assembly processes, even more important than environmental variation (i.e.*,* habitat filtering). However, a bond must exist between community phylogenetic structure and the performance of each species, a fact that is largely overlooked. The fitness of particular species might consequently be explained by the composition of the community in which the species is found. Furthermore, Ricklefs (2004) reported that community level processes generated population level changes, driving current ecological patterns. Yang et al. (2013) evaluated the phylogenetic diversity (PD) of assemblages surrounding target individuals, showing that most individuals had a neutral tendency regarding the PD of adjacent plots. However, by exploring the phylogenetic distance of each individual in a focal species, the direct effect of the surrounding community structure may be measured. For instance, Jiang et al. (2010) designed an experiment to assess the success of an invader species in bacterial communities, based on phylogenetic distance between invader and native species. The authors found a positive relationship between phylogenetic distance and the probability of a species becoming established.

The central role of biotic interactions is considered contingent on a species geographic range. For example, Brown et al. (1996) reported that biotic interactions tend to limit the distribution and abundance of species at lower latitudes. Differences in a species geographic range size do not just suggest variation in response to environmental variables or niche breadth (Gaston and Spicer 2001), but can reflect a species response to biologic interactions, which were illustrated in classic experiments by Connell (1983), and more recent studies by Bullock et al. (2000). However, previous studies reported the influence of biologic interactions in two-species systems distributions, without evaluating the effects of the entire community. Recently, Villalobos et al. (2013) introduced a novel and interesting approach, in which the phylogenetic structure of species co-occurrence of a focal species is used to study broad coexistence patterns.

We hypothesize that the sensitivity of species confronting negative interactions is reflected in the species population attributes: species inhabiting different assemblages, consequently experience different levels of competition throughout the species geographic range, which results in different rates of change in a species overall fitness. Gaston (2009) indicated that population size is the outcome of several population structure attributes (e.g., levels of births, deaths, and migration). For instance, population density has been linked to species richness, producing higher density in areas where richness is low (i.e.*,* density compensation; MacArthur et al. 1972), which is a pattern that was first described for island systems compared with mainland systems. Under these conditions, it is expected that populations of a species that occupy large geographic ranges (widespread) are not as influenced by co-occurring species with which they compete; alternatively, populations of species exhibiting restricted geographic ranges are more affected by increased potential competition. Support for range size heritability (Waldron 2007; but also see Webb and Gaston 2005) facilitates the expectation that related species would have similar range size; however, differences in the range of closely related species might serve as a viable system to test whether this dissimilarity is provided by a differential response to competition. The present study included two components: (1) we modified a widely used metric of phylogenetic structure (NRI; Webb et al. 2002) to center the attention toward a focal species and (2) two sympatric species in the genus *Peucaea* (Emberizidae), which exhibit very dissimilar range sizes, were used to evaluate whether a population size response to potential competition (through the modified metric) differs between the two sparrow species.

## Methods

### Peucaea sparrows and fieldwork

We conducted this study in southeast Mexico, in a region called the Tehuantepec Isthmus (Huidobro et al. 2006). This region is located in the narrowest stretch of land between the Gulf of Mexico and the Pacific Ocean, which is represented by the municipality of Juchitán, state of Oaxaca. Two sparrow species co-occur in the area: *Peucaea ruficauda* and *P. sumichrasti*. The former is a more widespread species (2.6 × 10^5^ km^2^; see next section) compared with *P. sumichrasti*, which is one of the most range-restricted avian species in Mexico (9.7 × 10^3^ km^2^; Wolf 1977). This species is endemic to the Tehuantepec Isthmus, and its entire range overlaps with *P. ruficauda*. Both sparrow species are common birds in the region and are similar in shape, behavior, and ecology (Wolf 1977). We selected 17 monitoring sites across the region, which had comparable vegetation type, human perturbation, and size, covering almost the entire geographic range of *P. sumichrasti*. The species composition (land birds) of each assemblage and *Peucaea* sparrow abundance were described by sampling 24 fixed-radius point counts separated by 200 m to avoid double counting individuals at each monitoring site. Each point count was sampled eight times by the same team of observers for one year, with each monitoring site being visited every 6 weeks on average.

### Phylogeny and geographic range size

We conducted a phylogenetic reconstruction, which included all land bird species we observed during our fieldwork. We queried the GenBank database (NCBI, September–October 2011) for the mitochondrial COI gene (Hebert et al. 2003; Alif et al. 2011; Appendix A1), representing each species we identified from our 17 monitoring sites. Sequences of the species not deposited in GenBank were replaced with an available sequence from the closest relative, according to previous studies reported in the literature. Sequence alignments were made in Clustal W (Larkin et al. 2007), a BioEdit 7.0.9.0 (Hall 1999) accessory tool. The reconstruction was performed by Bayesian method using BEAST v1.7.5 (Drummond and Rambaut 2007). Nucleotide substitution model employed was HKY+G+I being identified as the more adequate by JModelTest 0.1.1. The Yule speciation process was set to model the tree prior. In order to calibrate the root node of the tree, we used the date from Jetz et al. (2012) for the divergence of Anatidae (lognormal mean 4.27, SD 1, zero offset 0). One independent 5,000,000 generation run was performed sampling at every 1000 generations. The outcome was analyzed in TreeAnnotator v1.7.5 discarding the 10% of trees and visualized in Mesquite v2.74 (Maddison and Maddison 2011) and is available through Figshare (http://dx.doi.org/10.6084/m9.figshare.865723).

We looked for range size phylogenetic signal as an Emberizidae family trait by conducting a randomization test (999 randomizations; Blomberg et al. 2003) using the comprehensive phylogeny of Emberizidae reported by DaCosta et al. (2009), and the range size of 49 sparrow species. The species distributions area calculations were generated in ArcGIS 10 and shapefiles from the NatureServe compilation (Ridgely et al. 2003) using the appropriate geographic coordinates regarding species distributions in North or Central America. Phylogenetic signal is detected when random distributions exhibit significant differences from observed values. Although similar geographic range sizes have been observed among close relatives in birds (Waldron 2007), we decided to test this hypothesis in the Emberizidae family because the outcome was scale dependent.

### Data analysis

**Figure 1:**
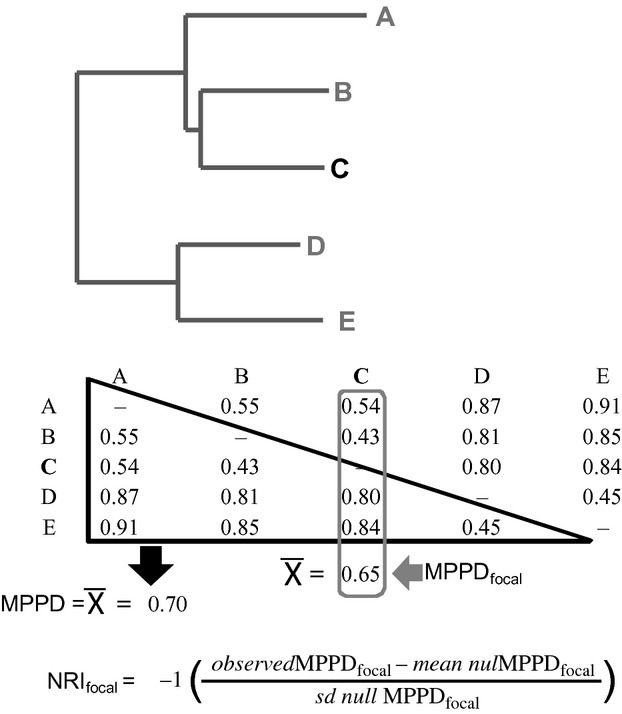
The NRI_focal_ calculation using a phylogenetic tree and its phylogenetic distance matrix. Notice that the difference with traditional NRI appears in the MPPD calculation, where the traditional is estimated by averaging the distances among all the possible pairs, while MPPD_focal_ is estimated by averaging the distances among the focal species (species C in this example) and each species included in the assemblage.

The traditional metric of the phylogenetic community (Net Relatedness Index -NRI-) is a standardized measure of the mean pairwise phylogenetic distance (MPPD, Webb et al. 2002), which is the phylogenetic distance among all possible pairs of species within a community. We modified the MPPD, so that our metric (MPPD_focal_) did not reflect the distance among all pairs, rather it measured the distance from the focal species and each species included in the assemblage, and averaged those values (Fig. 1). The calculation was performed using the cophenetic distance between focal species and all others members of the community, from which the mean was calculated. The species abundances are included in the weighed version of this metric by calculating the weighed mean instead of the arithmetic mean; in this way, the metric reflects the real composition of the community. Like the NRI, we constructed a standardization named NRI_focal_, in which the observed MPPD_focal_ values were compared with null distributions that were generated by creating communities of identical size by random draws from species pool (Kraft et al. 2007). In a single value, the NRI_focal_ describes focal species relatedness and the set of species that co-occur with the focal species. In this way, NRI_focal_ may be defined as the phylogenetic (ecological) similarity of *Peucaea* with all other observed bird species and serves as a measure of potential competition (Fig. 1).

We calculated NRI_focal_ (both weighed and non-weighed) for *P. ruficauda* and *P. sumichrasti* for each assemblage and performed a Pearson′s product-moment correlation between NRI_focal_ values and sparrow abundance. *Peucaea* abundance was normalized by root square transformation (Sokal and Rohlf 1987). The modified metric was based on the “mpd” function implemented in the PICANTE package (Kembel et al. 2010; in R 2.15.1 R Development Core Team 2010). The relationship of NRI_focal_ and NRI was calculated through 100 simulated communities, measuring both the phylogenetic metrics of each assemblage and calculating the correlation between them.

## Results and Discussion

**Figure 2:**
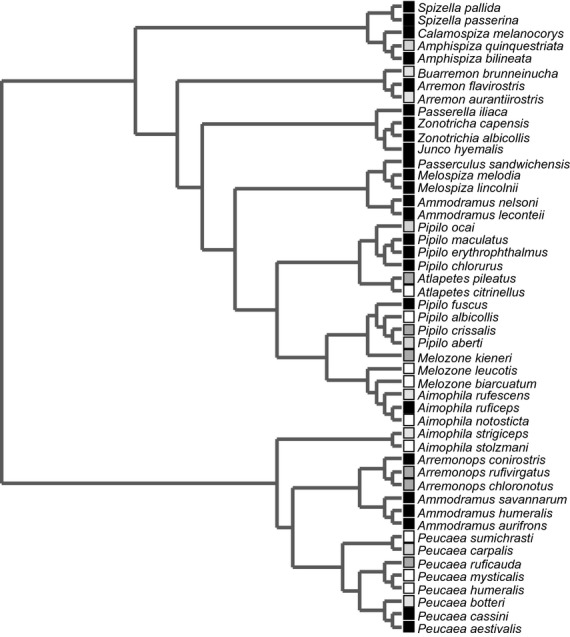
Geographic range size among Emberizidae sparrows showed a phylogenetic signal suggesting that closely related species have similar range sizes. Color indicates the size of geographic range (in thousands of square kilometers) for each species. White = 1–100; light gray = 101–200; dark gray = 201–500; slate gray = 501–1000; black <1000.

**Figure 3:**
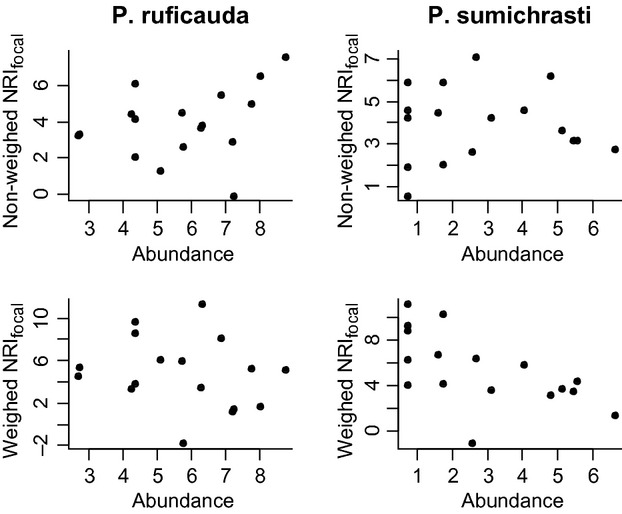
Scatter plot for correlation analysis between NRI_focal_ and *Peucaea* abundances (root square transformation). The species abundances are included in the weighed version of NRI_focal_ by calculating the weighed mean instead of the arithmetic mean.

The geographic range among Emberizidae sparrows represents a trait with significant phylogenetic signal (P < 0.001), indicating that similar species have a similar range size (Fig. 2). However, the difference in the geographic range size of sympatric and closely related *Peucaea* sparrows is noticeably large (2.5 × 10^5^ km^2^). This range size difference is of interest because of the ecological similarity between the sparrow species and the apparent lack of any form of geographic barrier; hence, this scenario may be explained by biologic interactions. We found that the widespread species (*P. ruficauda*) was more abundant compared with *P. sumichrasti* in 80% of the species assemblages. The relationship between range size and local abundance has been well established in several taxa and was demonstrated in passerine birds (Bock and Ricklefs 1983). Several mechanisms have been proposed to determine the positive correlation between range size and local abundance (revised and discussed by Gaston et al. 1997); for example, Holt et al. (1997) conferred special importance to among-species differences based on species differential responses to density-independent factors influencing population attributes. Here, results emphasized that biologic interactions were an important element in this process. For the resident species assemblages, the number of *P. sumichrasti* individuals showed a negative correlation with NRI_focal_ (r = −0.592, P < 0.05; Fig. 3) when considering all species abundances (weighed NRI_focal_). This suggests that competition strength (implied by increasing phylogenetic similarity) limits abundance in *P. sumichrasti*. The competition strength approached by the phylogenetic similarity leads the population volume in this restricted-sized species. Consequently, the community structure (composition of the species assemblage) is an important factor affecting one species population size. This result is analogous with that reported by Jiang et al. (2010), who found a positive relationship between the phylogenetic distance of invaders to bacterial communities and invader abundance. While the context of this preceding study differs to ours, it is worth noting the similar outcomes, even for very different organisms and differently sized communities. However, we did not find evidence of a relationship between widespread *P. ruficauda* abundance and NRI_focal_ (Table 1). Dissimilarity in range size among ecologically similar species might be related to differences in sensitivity to competition among species. This difference might be linked to the categories defined by Yang et al. (2013) in a lightly similar context, who grouped phylogenetic diversity into neutral, repeller, and accumulators species. Sensitivity to competition as a trait has the potential to limit species distributions across large geographic regions, because a large area exhibits high species turnover; consequently, several communities may form levels of competition so great that a sensitive species could not compete successfully. Consequently, regions at low latitudes with high species turnover can be defiant to susceptible species producing small-sized range species and contributing (besides other factors; Arita et al. 2005) to the observed and proved pattern of Rapoport′s rule. Yang et al. (2013) identified only a few phylogenetic diversity repellers, which might indicate that few sensitive species produce a reduced number of species with small-sized ranges and elevated numbers of species with large-sized ranges. However, the opposite pattern is obtained in reality; whereby, many species have small- to moderate-sized ranges, while only a few have very large ranges (Brown et al. 1996). Thus, the complex interaction between biologic interactions and environmental variation is reaffirmed.

**Table 1:** Correlation test results between *Peucaea* sparrow population sizes and our “focal” version of Net Relatedness Index (NRI_focal_) from 17 bird assemblages. The relative abundances from each entire assemblage were used to weigh the NRI_focal_. Note the negative relationships represent an increase between population size and phylogenetic dissimilarity.

	Non-weighed NRI_focal_		Weighed NRI_focal_	
	*r*	P-value	*r*	P-value
*Peucaea ruficauda*	−0.309	0.227	0.2	0.439
*Peucaea sumichrasti*	−0.004	0.987	−0.592	**0.012**

**Figure 4:**
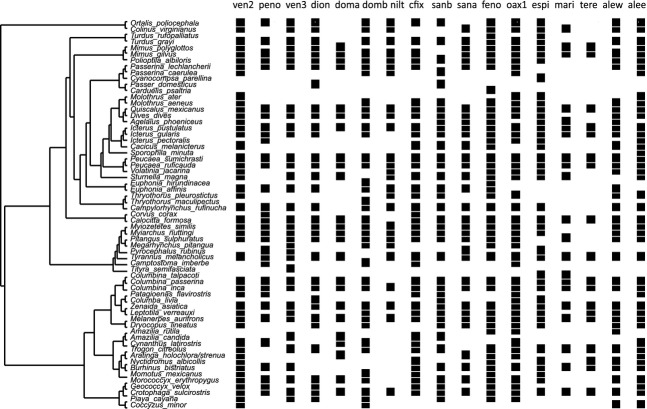
Reconstruction of a phylogeny of the species included in the study and their presence at the 17 monitoring sites.

The internal distributional range structure (sensu Brown et al. 1996), and the variability in species abundance throughout a species distribution (Brown et al. 1995), might provide a way of elucidating the response of species to potential competition across different assemblages. In even a relatively small area (Tehuantepec Isthmus), the observed sites showed enough species turnover (see Appendix A2) to depict dissimilar phylogenetically structured assemblages, which suggested a gradient of potential competition (Fig. 4). Moreover, the geographic boundaries of sensitive species might have been established by communities in which ecological (i.e.*,* phylogenetic) similarity exceeds the level of similarity (i.e., competition) that a focal species could withstand. In other words, certain assemblages function as “stakes,” limiting species geographic ranges. These stakes acted as biologic barriers setting a threshold that could not be trespassed by sensitive species. For instance, the *P. sumichrasti* population size reached low levels when the community phylogenetic similarity was high, preventing the dispersal of individuals to new areas. Although some individuals might cross these stakes, their numbers might be reduced, due to their being unable to establish stable populations. Areas where phylogenetic similarity is low would facilitate the free transit of sensitive species, until another type of barrier (i.e., environmental or biologic) is reached. Even though our monitoring sites were distributed throughout the geographic range of the restricted species, it would have been more accurate to evaluate our hypothesis by identifying the exact boundary of the range to assess the presence of overwhelming competition to which sensitive species are subjected. These boundaries are not distinct for birds; however, it might be viable to apply such studies that exhibit lower levels of movement to improve the experimental design.

In our study, the specific assemblage composition alone was not enough to explain the variability in population size among sites; the positive relationship between *P. sumichrasti* abundance and the nonweighted NRI_focal_ was not statistically supported (P > 0.05). Communities might appear similar to each other when species' abundances are neglected. In the Appendix (A2), we show that the dissimilarity among communities was low when only taking species composition into account, but become highly dissimilar when abundance was taken into account. Thus, our findings are based on species turnover, which is only evident when it is analyzed through abundance. Individuals of one species interacted with individuals of several species, and the level of competition differed in each interaction. We should think that individuals from one species are interacting with individuals of other species, rather than assuming that individuals interact with species; thus, abundances should be included in the analysis of phylogenetic structure whenever such data are available (Norden et al. 2012).Therefore, competition intensity among organisms must be weighed by phylogenetic distance. Pragmatically, when the scale and data are appropriate, patterns and processes are, respectively, revealed and inferred by weighing phylogenetic distance by the number of individuals.

The analyses were conducted using *Peucaea* population size as the species response; nonetheless, competition strength represented by the entire community could affect other population parameters, including birth, recruitment, and fecundity rates, among others. Gaston (2009) reviewed the population structure attributes necessary as a framework to continue research on the effects of community strength on population range dynamics, and methods similar to the present study show promise. The phylogenetic community ecology approach as a means of studying a single species clarifies patterns and processes that otherwise might be masked. Although NRI_focal_ and NRI are related methodologies (r = 0.626, P < 0.05), the metrics told different stories about the same assemblages. The NRI_focal_ we applied here provided a new method to evaluate the potential community effect over the focal species. Both metrics are superficially similar and derived from the same theoretical framework; nonetheless, we caution that metric application and results differ and should be applied under different contexts.

**Table 2:** Values of NRI_focal_, species richness, and abundances of *Peucaea* sparrows for each monitoring site.

		*Peucaea sumichrasti*			*Peucaea ruficauda*		
Site	Richness	Weighed NRI_focal_	Non-weighed NRI_focal_	Abundance	Weighed NRI_focal_	Non-weighed NRI_focal_	Abundance
ven2	47	−10.28	−5.94	3	−9.71	−6.12	18
peno	33	−1.46	−2.76	43	−1.26	−2.90	51
ven3	36	1.02	−2.66	6	1.73	−2.68	32
dion	34	−3.55	−3.19	29	−4.57	−3.29	7
doma	24	−4.25	−2.08	3	−3.90	−2.08	18
domb	38	−4.41	−3.21	30	−5.46	−3.36	7
nilt	27	−8.84	−4.63	0	−8.62	−4.15	18
cfix	40	−3.82	−3.67	26	−3.48	−3.68	39
sanb	39	−11.19	−4.25	0	−11.36	−3.84	39
sana	33	−6.75	−4.51	2	−6.04	−4.54	32
feno	48	−6.42	−7.14	7	−5.22	−7.62	75
oax1	46	−5.85	−4.63	16	−5.35	−5.00	59
espi	42	−9.29	−5.96	0	−8.11	−5.51	46
mari	21	−6.28	−1.92	0	−6.16	−1.35	25
tere	18	−4.06	−0.60	0	−1.47	0.04	52
alew	38	−3.70	−4.28	9	−3.35	−4.44	17
alee	45	−3.27	−6.22	22	−1.69	−6.52	63

## Appendix

**Appendix A1:** Accesion numbers from GenBank for the species included in the analysis.

*Peucaea ruficauda*	JQ173934
*Peucaea sumichrasti*	DQ433291
*Agelaius phoeniceus*	HM033218
*Amazilia candida*	FJ027764
*Amazilia rutila*	EU442323
*Aratinga holochlora/strenua*	GU826182
*Burhinus bistriatus*	JQ174203
*Cacicus melanicterus*	JQ174231
*Calocitta formosa*	DQ433558
*Camptostoma imberbe*	DQ433420
*Campylorhynchus rufinucha*	DQ433425
*Carduelis psaltria*	JN801283
*Coccyzus minor*	JQ174483
*Columbina inca*	DQ433529
*Columba livia*	JF498761
*Columbina passerina*	JN850709
*Columbina talpacoti*	JQ174508
*Colinus virginianus*	DQ433524
*Corvus corax*	GU571837
*Crotophaga sulcirostris*	JN801306
*Cyanocompsa parellina*	FJ027473
*Cynanthus latirostris*	JN802021
*Dives dives*	DQ433634
*Dryocopus lineatus*	JQ174725
*Euphonia affinis*	EU442311
*Euphonia hirundinacea*	EU442315
*Geococcyx velox*	JN801317
*Icterus gularis*	DQ433697
*Icterus pectoralis*	EU442293
*Icterus pustulatus*	DQ433688
*Leptotila verreauxi*	FJ027748
*Megarhynchus pitangua*	JN801790
*Melanerpes aurifrons*	EU442305
*Mimus gilvus*	JN801810
*Mimus polyglottos*	JQ175389
*Molothrus aeneus*	DQ433807
*Molothrus ater*	HM033587
*Momotus mexicanus*	AY275849
*Morococcyx erythropygus*	AY274064
*Myiarchus nuttingi*	DQ433828
*Myiozetetes similis*	FJ027897
*Nyctidromus albicollis*	JN801344
*Ortalis poliocephala*	AF165496
*Passerina caerulea*	HM033638
*Passer domesticus*	JQ175684
*Passerina lechlancherii*	DQ433884
*Patagioenas flavirostris*	JN801347
*Piaya cayana*	JN801921
*Pitangus sulphuratus*	JN801364
*Polioptila albiloris*	JN801366
*Pyrocephalus rubinus*	JQ288212
*Quiscalus mexicanus*	EU442320
*Sporophila minuta*	JQ176257
*Sturnella magna*	DQ433226
*Thryothorus maculipectus*	HM033838
*Thryothorus pleurostictus*	HM208688
*Tityra semifasciata*	EU442296
*Trogon citreolus*	JN802065
*Turdus grayi*	JQ176566
*Turdus rufopalliatus*	HM033871
*Tyrannus melancholicus*	JN802081
*Volatinia jacarina*	JQ627357
*Zenaida asiatica*	JQ176675

**Appendix A2:** Dissimilarity among monitoring sites using Bray–Curtis index. Upper diagonal reflects dissimilarity through qualitative data; meanwhile, lower diagonal is showing differences using abundances.

	ven2	peno	ven3	dion	doma	domb	nilt	cfix	sanb	sana	feno	oax1	espi	mari	tere	alew	alee
ven2	–	0.259	0.247	0.220	0.361	0.209	0.324	0.136	0.186	0.210	0.125	0.137	0.213	0.441	0.477	0.140	0.075
peno	0.677	–	0.229	0.224	0.298	0.268	0.322	0.151	0.296	0.242	0.309	0.250	0.351	0.472	0.480	0.268	0.256
ven3	0.610	0.492	–	0.155	0.279	0.227	0.270	0.221	0.253	0.171	0.224	0.214	0.282	0.368	0.407	0.227	0.268
dion	0.771	0.419	0.502	–	0.276	0.222	0.267	0.243	0.194	0.194	0.220	0.210	0.280	0.370	0.451	0.222	0.241
doma	0.874	0.658	0.642	0.560	–	0.387	0.360	0.313	0.419	0.228	0.389	0.352	0.446	0.364	0.415	0.387	0.333
domb	0.763	0.417	0.461	0.304	0.622	–	0.313	0.231	0.289	0.296	0.233	0.224	0.342	0.517	0.527	0.211	0.253
nilt	0.811	0.722	0.685	0.617	0.390	0.674	–	0.333	0.344	0.322	0.324	0.315	0.403	0.478	0.581	0.281	0.324
cfix	0.676	0.244	0.375	0.343	0.569	0.312	0.634	–	0.179	0.260	0.205	0.218	0.284	0.467	0.439	0.179	0.176
sanb	0.656	0.559	0.576	0.626	0.655	0.624	0.506	0.461	–	0.268	0.233	0.224	0.266	0.448	0.491	0.211	0.229
sana	0.756	0.563	0.558	0.572	0.487	0.618	0.421	0.472	0.395	–	0.210	0.275	0.243	0.321	0.400	0.183	0.231
feno	0.545	0.434	0.534	0.563	0.699	0.540	0.633	0.413	0.451	0.420	–	0.158	0.191	0.441	0.477	0.140	0.161
oax1	0.463	0.441	0.521	0.570	0.729	0.562	0.612	0.415	0.449	0.543	0.299	–	0.250	0.463	0.500	0.224	0.109
espi	0.735	0.671	0.691	0.678	0.702	0.688	0.556	0.627	0.512	0.545	0.483	0.546	–	0.377	0.414	0.215	0.209
mari	0.834	0.731	0.719	0.706	0.482	0.748	0.365	0.664	0.625	0.457	0.654	0.677	0.597	–	0.189	0.414	0.446
tere	0.886	0.647	0.687	0.671	0.492	0.733	0.666	0.590	0.754	0.622	0.737	0.790	0.733	0.385	–	0.418	0.452
alew	0.818	0.506	0.566	0.421	0.388	0.527	0.493	0.403	0.611	0.499	0.606	0.638	0.665	0.567	0.526	–	0.157
alee	0.634	0.327	0.464	0.356	0.637	0.432	0.681	0.242	0.502	0.485	0.347	0.356	0.592	0.689	0.629	0.461	–

## References

[b1] Alif LA, Khan HA, Shobrak M, Williams J (2011). Cytochrome c oxidase subunit I barcoding of the green bee-eater (*Merops orientalis)*. Genet. Mol. Res.

[b2] Arita HT, Rodríguez P, Vázquez-Domínguez E (2005). Continental and regional ranges of North American mammals: Rapoport′s rule in real and null worlds. J. Biogeogr.

[b3] Blomberg SP, Garland T, Ives AR (2003). Testing for phylogenetic signal in comparative data: behavioral traits are more labile. Evolution.

[b4] Bock CE, Ricklefs RE (1983). Range size and local abundance of some North American songbirds: a positive correlation. Am. Nat.

[b5] Brown JH, Mehlman DW, Stevens GC (1995). Spatial variation in abundance. Ecology.

[b6] Brown JH, Stevens GC, Kaufman DM (1996). The geographical range: size, shape, boundaries, and internal structure. Annu. Rev. Ecol. Syst.

[b7] Bullock JM, Edwards RT, Carey PD, Rose RJ (2000). Geographical separation of two Ulex species at three spatial scales: does competition limit species' ranges?. Ecography.

[b8] Burns JH, Strauss SY (2011). More closely related species are more ecologically similar in an experimental test. Proc. Natl Acad. Sci. USA.

[b9] Cadotte MW, Dinnage R, Tilman D (2012). Phylogenetic diversity promotes ecosystem stability. Ecology.

[b10] Connell JH (1983). On the prevalence and relative importance of interspecific competition: evidence from field experiments. Am. Nat.

[b11] DaCosta JM, Spellman GM, Escalante P, Klicka J (2009). A molecular systematic revision of the two historically problematic songbird clades: *Aimophila* and *Pipilo*. J. Avian Biol.

[b12] Drummond AJ, Rambaut A (2007). BEAST: Bayesian evolutionary analysis by sampling trees. BMC Evol. Biol.

[b13] Gaston KJ (2009). Geographic range limits: achieving synthesis. Proceed. Royal Soc. B-Biol. Sci.

[b14] Gaston KJ, Spicer JI (2001). The relationship between range size and niche breadth: a test using five species of *Gammarus* (Amphipoda). Glob. Ecol. Biogeogr.

[b15] Gaston KJ, Blackburn TM, Lawton JH (1997). Interspecific abundance-range size relationships: an appraisal of mechanisms. J. Anim. Ecol.

[b16] Gómez JP, Bravo GA, Brumfield RT, Tello JG, Cadena CD (2010). A phylogenetic approach to disentangling the role of competition and hábitat filtering in community assembly of Neotropical forest birds. J. Anim. Ecol.

[b17] Graham CH, Parra JL, Rahbek C, McGuire JA (2009). Phylogenetic structure in tropical hummingbird communities. Proc. Natl. Acad. Sci. USA.

[b18] Hall TA (1999). BioEdit: a user-friendly biological sequence alignment editor and analysis program for Windows 95/98/NT. Nucleic Acids Symp. Ser.

[b19] Hebert PDN, Ratnasingham S, de Waard JR (2003). Barcoding animal life: cytochrome c oxidase subunit 1 divergences among closely related species. Proceed. Royal Soc. B-Biol. Sci.

[b20] Holt RD, Lawton JH, Gaston KJ, Blackburn TM (1997). On the relationship between range size and local abundance: back to basics. Oikos.

[b21] Houle A (1997). The role of phylogeny and behavioral competition in the evolution of coexistence among primates. Can. J. Zool.

[b22] Huidobro L, Morrone JJ, Villalobos JL, Álvarez F (2006). Distributional patterns of freshwater taxa (fishes, crustaceans and plants) from the Mexican Transition Zone. J. Biogreog.

[b23] Jetz W, Thomas GH, Joy JB, Hartmann K, Mooers AO (2012). The global diversity of birds in space and time. Nature.

[b24] Jiang L, Tan J, Pu Z (2010). An experimental test of Darwin′s naturalization hypothesis. Am. Nat.

[b25] Kembel SW, Cowan PD, Helmus MR, Cornwell WK, Morlon H, Ackerly DD (2010). Picante: R tools for integrating phylogenies and ecology. Bioinformatics.

[b26] Kraft NJB, Cornwell WK, Webb CO, Ackerly DD (2007). Trait evolution, community assembly, and the phylogenetic structure of ecological communities. Am. Nat.

[b27] Larkin MA, Blackshields G, Brown NP, Chenna R, McGettigan PA, McWilliam H (2007). ClustalW and ClustalX version 2. Bioinformatics.

[b501] Lovette IJ, Hochachka WM (2006). Simultaneous effects of phylogenetic niche conservatism and competition on avian community structure. Ecology.

[b28] MacArthur RH, Diamond JH, Karr JR (1972). Density compensation in island faunas. Ecology.

[b29] Maddison WP, Maddison DR (2011). http://mesquiteproject.org.

[b30] Norden N, Letcher SG, Boukili V, Swenson NG, Chazdon R (2012). Demographic drivers of successional changes in phylogenetic structure across life-history stages in plant communities. Ecology.

[b31] R Development Core Team (2010). http://www.R-project.org/.

[b32] Ricklefs RE (2004). A comprehensive framework for global patterns in biodiversity. Ecol. Lett.

[b33] Ridgely RS, Allnutt TF, Brooks T, McNicol DK, Mehlman DW, Young BE (2003). http://www.natureserve.org/.

[b34] Sokal RR, Rohlf FJ (1987). Introduction to biostatistics.

[b35] Villalobos F, Rangel TF, Diniz-Filho JAF (2013). Phylogenetic fields of species: cross-species patterns of phylogenetic structure and geographical coexistence. Proceed. Royal Soc. B.

[b36] Waldron A (2007). Null models of geographic range size evolution reaffirm its heritability. Am. Nat.

[b37] Webb TJ, Gaston KJ (2005). Heritability of geographic range sizes revisited: a reply to Hunt. Am. Nat.

[b38] Webb CO, Ackerly DD, McPeek MA, Donoghue MJ (2002). Phylogenies and community ecology. Annu. Rev. Ecol. Syst.

[b39] Wiens JJ, Ackerly DD, Allen AP, Anacker BL, Buckley LB, Cornell HV (2010). Niche conservatism as an emerging principle in ecology and conservation biology. Ecol. Lett.

[b40] Willis CG, Ruhfel B, Primack RB, Miller-Rushing AJ, Davis CC (2008). Phylogenetic patterns of species loss in Thoreau′s woods are driven by climate change. Proc. Natl Acad. Sci. USA.

[b41] Wolf LL (1977). Species relationship in the avian genus *Aimophila*. Ornithol. Monog.

[b42] Yang J, Swenson NG, Cao M, Chuyong GB, Ewango CEN, Howe R (2013). A phylogenetic perspective on the individual species-area relationship in temperate and tropical tree communities. PLoS ONE.

